# Model Analysis of Digital Models in Moderate to Severe Crowding: In Vivo Validation and Clinical Application

**DOI:** 10.1155/2018/8414605

**Published:** 2018-01-14

**Authors:** Jae Hee Yoon, Hyung-Seog Yu, Yoonjeong Choi, Tae-Hyun Choi, Sung-Hwan Choi, Jung-Yul Cha

**Affiliations:** ^1^Department of Orthodontics, College of Dentistry, Yonsei University, Seoul, Republic of Korea; ^2^Department of Orthodontics, Institute of Craniofacial Deformity, College of Dentistry, Yonsei University, Seoul, Republic of Korea; ^3^Department of Dentistry, Seoul National University Bundang Hospital, Seongnam, Republic of Korea

## Abstract

**Objective:**

We investigated the suitability of intraoral-scan models for measuring tooth dimensions and the amount of crowding in patients with severe tooth crowding.

**Materials and Methods:**

Fifty-eight patients who had undergone intraoral scans for diagnosis were included. Cast models were divided into two groups depending on the amount of crowding, as determined by initial caliper-based measurements (mild crowding [MC] group: <3.0 mm; severe crowding [SC] group: >4.5 mm). Twenty maxillary models and 20 mandibular models were used in this study. For the three types of models (i.e., IS digital model, C cast model, and CS digital model), the reproducibility and the precision of linear measurements were evaluated.

**Results:**

We found that linear measurements made using digital calipers on a plaster model and on the relevant software were reproducible. There was no significant difference in most linear measurements between digital models and the C model. There were differences in the amount of crowding (*p* < .05), although these were not clinically significant. There was no relationship between the precision of crowding in the three types of models and the severity of crowding.

**Conclusions:**

Digital models can be used for measuring crowding in both mild and severe crowding cases. However, crowding measured by digital models tends to be lesser than that measured by cast models, and this should be considered during clinical application.

## 1. Introduction

The accuracy of dental model analysis is essential for using digital models as diagnostic tools [1]. Recently, greater accuracy has been required for 3D digital models, as they are used not only for diagnosis but also for planning treatments and for the fabrication of orthodontic appliances [2].

Many studies have assessed the accuracy of digital models, of which cast models are the gold standard, for making orthodontic diagnoses and linear measurements [3, 4]. For linear measurements of tooth size, the mean differences in tooth dimension varied from 0.01 to 0.45 mm between the models [5, 6]. For measurement of mild tooth crowding, the difference between digital and cast models ranged from 0.19 to 1.19 mm for the digital model [4, 6]. However, only two studies included samples with different amounts of crowding, and they found an increased discrepancy, up to 3 mm, between the digital and plaster models, due to an accumulation of measurement error during the space analysis. The authors speculated that the inaccuracy of digital analysis was due to the difficulty of locating the proper mesiodistal width for the space analysis [4, 7].

In addition, some studies have evaluated the scanning time and the accuracy of orthodontic diagnoses made using intraoral scans [8, 9]. Grünheid et al. [8] suggested that there were no significant differences between cast-scan models and intraoral-scan models when they were digitally superimposed and the surface areas were compared. Furthermore, Wiranto et al. [9] compared intraoral-scan models, cast models, and cone-beam computed tomography scans of cast models in patients who had mild to moderate crowding and suggested that tooth size measurements and the Bolton ratio of digital models could be used in patients with mild to moderate crowding.

Recently, some studies have used reference models to assess the accuracy of intraoral scanning in evaluating the precision and trueness of the total arch scan model [10, 11]. They concluded that intraoral scanning produced clinically acceptable results. However, intraoral-scan models taken from real patients might be less accurate, due to patient movement, limited intraoral space, intraoral humidity, and saliva flow [12]. Further studies are needed to validate the accuracy of intraoral scanning in real patients.

Given this background, it is clear that further studies are needed to assess the use of intraoral-scan digital models for complex diagnostic cast analyses, such as crowding analysis, particularly in real patients with severe crowding. The purpose of this study was to assess the relationship between the accuracy of linear measurements and crowding by comparing intraoral-scan models, cast-scan models, and cast models of patients with various degrees of crowding.

## 2. Materials and Methods

Approval for this study was obtained from the institutional review board of #####. One hundred and forty-three sets of maxillary and mandibular pretreatment intraoral-scan impressions of patients who visited the orthodontic clinic at #####, from December 2013 to April 2016, were used for this study. The inclusion criteria were as follows: (1) absence of dentofacial deformity or medical problems; (2) absence of previous orthodontic history with fixed appliances; (3) eruption of all permanent teeth, without any impacted, missing, or supernumerary teeth, from one of the first molars to the next first molar; (4) availability of an intraoral-scan model and a cast model.

In total, 46 patients were included in this study. Before measuring the amount of crowding, linear measurements of overjet and overbite were performed for the 46 pairs of models of 46 patients. To highlight the contrast in severity, samples were divided into two groups, depending on the amount of crowding as determined by initial caliper-based measurements. Cast (C) models in cases where the amount of crowding was less than 3.0 mm were included in the mild crowding (MC) group (maxilla [Mx]: *n* = 20, mandible [Mn]: *n* = 20), and those with more than 4.5 mm were included in the moderate to severe crowding (SC) group (Mx: *n* = 20, Mn: *n* = 20). Six upper arches and 6 lower arches were excluded because of the amounts of crowding were not matched to features of both mild and severe group. Linear measurements of arch width, arch length, tooth width, and sectional arch length were performed in the 80 arches.

Since it is not ethically and clinically possible to obtain linear measurements of the width of actual patients' teeth [13], C models and two types of digital models (intraoral- and cast-scan) were analyzed and compared in this study. Intraoral-scan (IS) models were obtained for diagnosis using an intraoral scanner (TRIOS 3; 3Shape, Copenhagen, Denmark) according to the manufacturer's protocol. Alginate (Cavex CA37; CAVEX, Haarlem, Holland) impressions were taken from patients who were scheduled for treatment with indirect bonding. Wax bite registration was performed with centric occlusion in an upright position. C models were made in the standard way using pouring plaster (Rhombstone White; Ryoka Dental, Mie-Ken, Japan). All C models were scanned using the Orapix 3D dental system (Orapix, Seoul, Korea), yielding the cast-scan models (CS). All digital models were 3D oriented and model data were saved as stereolithographic (STL) files.

Linear measurements of the C models were performed using a digital caliper (Fisher Scientific International Inc., Hampton, NH, USA), which had an accuracy of 1/100 mm. Linear values of the digital models were measured with a Rapidform XOR3 64 (Geomagic, Morrisville, NC, USA) and saved to an accuracy of 1/100 mm. All of the linear measurements were performed twice by one operator (Jae Hee Yoon), with an interval of more than 1 week between assessments to confirm the reproducibility of the measurements.

**Figure 1:**
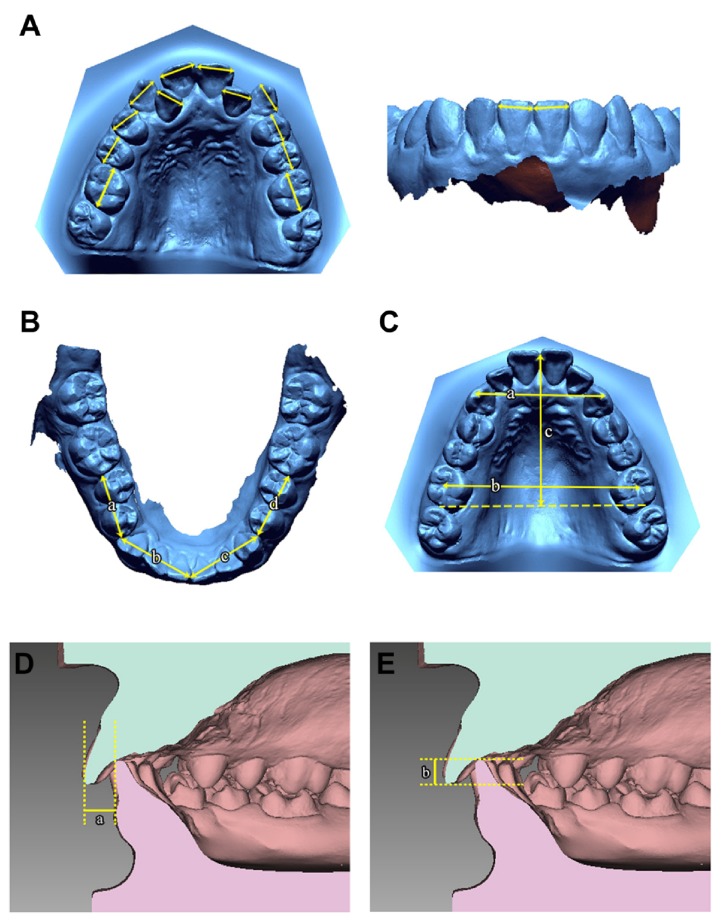
Linear measurements used in this study. (A) Tooth width measurement; the greatest width of the posterior tooth in the occlusal view; the greatest width of the anterior tooth in the labial view. (B) Sectional arch length was measured in four separate segments. a, d: posterior sectional arch length; length between the mesial contact point of the first molar and the distal contact point of the canine. b, c: anterior sectional arch length; length between the distal contact point of the canine and the mesial contact point of both central incisors. (C) a, b: arch width; the distance between the cusp tips of both canines and both first molars. c: arch length; the distance from the line connecting the distal surface of the first molars to the contact point between the central incisors. (D) a: overjet. (E) b: overbite.

The linear measurements used in this study are described in Figure 1. For the linear measurement of tooth width, the mesiodistal points representing greatest width of the posterior tooth were chosen from the occlusal view, while the mesiodistal points representing the greatest width of the anterior tooth were chosen from the labial view. Before measuring distance, it was confirmed that the lines connecting measuring points are parallel to central groove and perpendicular to the axis of measuring teeth in posterior teeth and the lines connecting measuring points are perpendicular to the axis of measuring teeth in anterior teeth.

Crowding was defined as follows: (the sum of the mesiodistal width of the second premolar to the opposite second premolar) − (the sum of the sectional arch length).

*Statistical Analysis*. Intraclass correlation coefficients (ICCs) were calculated to confirm the reproducibility of the measurements (0.96–1.00). After confirming reproducibility, the mean values of two measurements taken on both the Mx and Mn were calculated and compared for each linear measurement of a single C model and two digital models, using a repeated-measurements analysis of variance (ANOVA) (Bonferroni's method). Samples were divided into either the MC or SC group, depending on the severity of crowding; the precision of the linear measurements and the amount of crowding in the three kinds of models were compared by a repeated-measures ANOVA. Finally, the difference in the precision of measurement in each model between the MC and SC groups was confirmed using the Greenhouse-Geisser correction. All statistical analyses were performed using SPSS (version 21; SPSS, Chicago, IL, USA).

## 3. Results

The mean amount of crowding in the maxillary MC group and mandibular MC group was 1.23 mm (standard deviation [SD], 1.48 mm) and 1.47 mm (SD, 1.04 mm), respectively. The mean amount of crowding in the maxillary SC group and mandibular SC group was 6.57 mm (SD, 2.13 mm) and 6.68 mm (SD, 3.01 mm), respectively.

There was no significant difference in the values of overjet and overbite between the three models (Table 1). There was no significant difference in the arch width and arch length between the C and CS models or between the CS and IS models. However, there were some significant differences between the C and IS models in the arch length (AL) (*p* = .00) of the Mx. There were also significant differences in the intermolar width (AWM) (*p* = .03) and AL (*p* = .00) between C and IS models of the Mn (Table 2).

Comparisons of linear measurements between groups for maxillary dentition are presented in Table 3. There were significant differences in the mesiodistal widths of the maxillary first premolar (*p* = .00) and second premolar (*p* = .01), and in the amount of crowding (*p* = .01) between the C and IS models in the MC group. There was also a significant difference in the mesiodistal width of the first premolar (*p* = .01) between the CS and IS. In the SC group, there was a significant difference between the C and IS at the maxillary lateral incisor (*p* = .00) and first premolar (*p* = .01) and in the amount of crowding (*p* = .02).

The comparisons of linear measurements between groups for mandibular dentition are shown in Table 4. There were significant differences in the mesiodistal width of the mandibular first premolar (*p* = .02), second premolar (*p* = .01), and first molar (*p* = .00) between the C and CS models in the MC group. Furthermore, there were also significant differences in the mesiodistal width of the mandibular first premolar (*p* = .00), second premolar (*p* = .00), and first molar (*p* = .00) between the C and IS models in the MC group. In the SC group, there were significant differences between the C and IS scans in the mesiodistal width of the mandibular first premolar (*p* = .00) and second premolar (*p* = .00) and in the amount of crowding (*p* = .02). Only the mandibular second premolar (*p* = .00) in the MC group showed significant differences in width between the CS and IS models.

There was no interaction between severe crowding (more than 4.5 mm) and the precision of dental measurements in the three dental models (*p* > .05) (Table 5).

## 4. Discussion

To evaluate the validity of digital model analysis, IS models that were generated by scanning actual patients in clinical conditions were used in this study, unlike previous studies that mostly used reference models [4, 6]. In addition, patients with various degrees of crowding were scanned to confirm whether digital intraoral scanning is a sufficiently reliable method for use in orthodontic diagnoses in patients with severe crowding.

The reproducibility of both the caliper measurements and digital measurements was confirmed by calculating the ICCs. The ICC was 0.98–1.00 for the C models and 0.96–1.00 for the digital models, which was similar to those obtained in a previous study [14].

There were no significant differences in the measurements of overjet and overbite in the three models. Asquith and McIntyre [15] stated that errors of more than 0.5 mm are clinically unacceptable for overjet. Therefore, our findings suggest that it is appropriate to make diagnostic decisions based on values of overjet and overbite measured from digital models.

There was no significant difference in the arch width of canines on either the Mx or the Mn among the C models, CS models, and IS models; this finding corresponds with the results of the study by Reuschl et al. [2] However, there was a significant difference in arch length between C and IS in both the Mx and Mn, as well as in the intermolar width between the C and IS in the Mn. This may be explained by the arch distortion (value: <170 *μ*m) that occurs during the scanning process [10], which makes the arch width seem wider than it actually is in the molar area and causes it to be shifted forward into the incisal area.

The mean difference in the mesiodistal width in this study ranged from −0.02 mm to 0.12 mm, indicating that the errors in model analysis using a digital model were at a clinically acceptable level, compared with those of a previous study [13]. These differences between the digital models and C models could be explained by the following reasons.

First, the differences between the C model and IS model or CS model and IS model include the error that occurs when the C model is fabricated using an alginate impression. A suitable C model can be created only by pouring the plaster immediately after taking the impression [16, 17]; although we did this, distortion of the C model could not be completely controlled.

Second, the limited space and moisture in the mouth during the intraoral scanning process may have caused significant differences, especially in posterior teeth [12].

Third, there were differences in the measuring method between the C model and digital model. Unlike in the direct caliper-based measuring method, there is no physical barrier dictating the placement of the caliper on measuring points when using a digital model. Thus, as long as a careful measuring point is selected on the computer screen, it would be reasonable to believe that digital measurements are more valid than those made using calipers on plaster [13].

In the present study, the samples were divided according to the amount of crowding. In the SC group, there was a significant difference in the width of the upper lateral incisors between the C model and the IS model, unlike in the MC group. This may be due to the difficulty of reproducing the proximal surface of the tooth in a model with marked crowding during the process of reconstructing data into an STL file, where the inner part of the model is represented as a hollow object [18].

**Figure 2:**
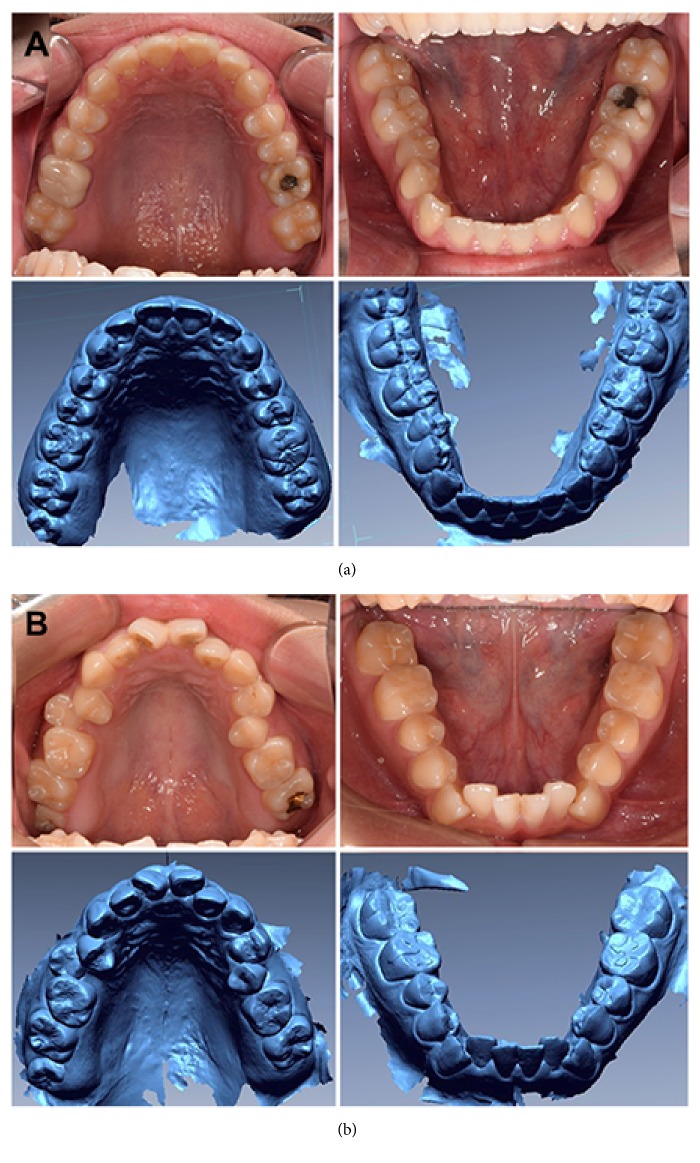
Comparison of intraoral scan model and real dentition. (a) Intraoral-scan model and intraoral photo of mild crowding. (b) Intraoral-scan model and intraoral photo of severe crowding. The proximal surface of the tooth is reproduced with acceptable quality in both mild and severe crowding; in some cases, it was easier to measure the mesiodistal width of posterior tooth in the severe crowding model than in the mild crowding model because of exposure of measuring points from posterior crowding.

In addition, there may have been more significant differences in the MC group than in the SC group in the posterior teeth because of difficulties in measuring the mesiodistal width of the teeth (Figure 2). In the MC group, the most distal points (or most mesial points) of the posterior teeth, where they make complete contact with adjacent teeth, are frequently selected as measuring points. In contrast, in the SC group, most measuring points were exposed because of mesial tipping or crowding, and, thus, error during measurement was reduced. In the anterior teeth, in contrast, there were a few errors in mild crowding group, so it was less effective than the exposure of the contact points by crowding. These results are in contrast to those of previous studies [4, 18]; thus, further studies should consider not only the total amount of crowding but also how crowding in specific locations affects the precision of linear measurements [19].

When determining the mean difference in the amount of crowding, cast analysis yielded higher values than digital analysis, except for maxillary measurements in the MC group. Im et al. [18] and Reuschl et al. [2] reported that the mesiodistal widths of most teeth were underestimated in a digital model compared to those in a C model. This tendency needs to be considered when a diagnosis is made based on digital models without a cast, as most clinicians are accustomed to diagnosing and establishing treatment plans based on C model analysis.

To evaluate the inaccuracy of measurements of tooth width in digital models with severe crowding, as mentioned in a previous study [4], the interaction effect of the severity of crowding was confirmed on the dental measurements in three dental models. There was no interaction between severe crowding (>4.5 mm) and the precision of dental measurements in the three dental models studied here.

Clearly, none of the three methods tested here can produce an exact replica of the patient's actual dentition. Instead of using a C model as the gold standard, as was done in a previous study [13], we tried to compare the diagnoses made using digital models with those made conventionally based on C models.

There were some differences in the precision of space analysis between different crowding groups, but these did not reach statistical significance in the interaction analyses. Thus, using an intraoral scanner for diagnostic purposes in the orthodontic clinic is appropriate and useful. Moreover, C models that have already been stored can be replaced with CS models for ease of storage. Further studies are needed to determine the effect of the state or distribution of crowding on the precision of linear measurements, by analyzing teeth in specific areas with severe crowding.

## 5. Conclusion

Linear measurements of both the C model and digital model were reproducible. Moreover, there was no significant difference in most linear measurements between the digital models and the C models. However, there were significant differences in the amount of crowding due to the accumulation of errors that occurred in single measurements. The differences in crowding were not dependent on the severity of crowding (>4.5 mm and <3.0 mm). In addition, the differences did not have a clinically meaningful effect (mean difference = −.02–.86 mm). Thus, as with C models, digital models can also be used for measuring crowding in severe crowding (>4.5 mm) cases. However, the use of digital models for cases of less crowding should be considered.

## Figures and Tables

**Table 1:** Mean overjet and overbite of cast model and 2 digital models (mm).

Measurement	Cast (C)	Cast scan (CS)	Intraoral scan (IS)	Mean deviation/	
	(mean ± SD)	(mean ± SD)	(mean ± SD)	standard error (*p* value)	
Overbite	0.73 ± 2.11	0.74 ± 2.01	0.77 ± 2.06	C-CS	−.01/.33 (1.00)
				C-IS	−.04/.09 (1.00)
				CS-IS	−.04/.33 (1.00)

Overjet	2.69 ± 2.87	2.59 ± 2.75	2.62 ± 2.79	C-CS	.11/.38 (1.00)
				C-IS	.07/.08 (1.00)
				CS-IS	−.04/.36 (1.00)

*p* values were calculated by RM-ANOVA.

**Table 2:** Mean linear measurements of arch width and arch length of cast model and two digital models.

	Cast (C)	Cast scan (CS)	Intraoral scan (IS)	Mean difference/	
	(mean ± SD)	(mean ± SD)	(mean ± SD)	standard error (*p *value)	
Maxilla					
AWC	35.25 ± 2.46	35.20 ± 2.56	34.99 ± 2.41	C-CS	.22/.39 (1.00)
				C-IS	.14/.09 (.45)
				CS-IS	−.08/.39 (1.00)
AWM	48.98 ± 3.75	48.84 ± 3.56	48.88 ± 3.90	C-CS	−.08/.43 (1.00)
				C-IS	−.13/.15 (1.00)
				CS-IS	−.06/.42 (1.00)
AL	40.87 ± 2.88	40.44 ± 2.81	39.28 ± 2.58	C-CS	.26/.53 (1.00)
				C-IS	1.35/.30 (.00)^*∗∗*^
				CS-IS	1.09/.66 (.23)

Mandible					
AWC	27.23 ± 2.86	27.20 ± 2.86	27.10 ± 2.84	C-CS	.03/.41 (1.00)
				C-IS	.13/.06 (.10)
				CS-IS	.10/.42 (1.00)
AWM	42.58 ± 3.11	42.13 ± 3.53	42.43 ± 3.22	C-CS	.45/.55 (1.00)
				C-IS	.15/.06 (.03)^*∗*^
				CS-IS	−.30/.55 (1.00)
AL	36.36 ± 2.46	36.33 ± 2.53	35.51 ± 2.14	C-CS	.14/.50 (1.00)
				C-IS	.95/.26 (.00)^*∗∗*^
				CS-IS	.81/.51 (.35)

AWC, intercanine width; AWM, intermolar width; AL, arch length (*n* = 40); *p* values were calculated by RM-ANOVA; ^*∗*^*p* < .05; ^*∗∗*^*p* < .005.

**Table 3:** Comparison of linear measurements between mild and severe crowding groups in maxillary dentition (mm).

	Cast (C)		Cast scan (CS)		Intraoral scan (IS)		Mean difference (*p* value)		
	(mean ± SD)		(mean ± SD)		(mean ± SD)				
	Mild group	Severe group	Mild group	Severe group	Mild group	Severe group		Mild group	Severe group
Mesiodistal width of central incisor	8.50 ± 0.46	8.87 ± 0.57	8.54 ± 0.49	8.82 ± 0.57	8.48 ± 0.47	8.82 ± 0.61	C-CS	−.04 (.59)	−.04 (1.00)
							C-IS	.02 (1.00)	.05 (.12)
							CS-IS	.06 (.31)	.01 (1.00)

Mesiodistal width of lateral incisor	7.16 ± 0.55	7.62 ± 0.47	7.12 ± 0.55	7.51 ± 0.49	7.17 ± 0.54	7.47 ± 0.49	C-CS	.05 (.26)	.10 (1.00)
							C-IS	−.00 (1.00)	.15 (.00)^*∗∗*^
							CS-IS	−.05 (.36)	.05 (1.00)

Mesiodistal width of canine	8.10 ± 0.38	8.21 ± 0.46	8.12 ± 0.39	8.20 ± 0.45	8.08 ± 0.42	8.22 ± 0.45	C-CS	−.02 (1.00)	.01 (1.00)
							C-IS	.02 (1.00)	−.01 (1.00)
							CS-IS	.38 (.44)	−.02 (1.00)

Mesiodistal width of first premolar	7.66 ± 0.42	7.87 ± 0.52	7.64 ± 0.39	7.84 ± 0.52	7.51 ± 0.43	7.78 ± 0.53	C-CS	.02 (1.00)	.03 (1.00)
							C-IS	.15 (.00)^*∗∗*^	.09 (.01)^*∗*^
							CS-IS	.13 (.01)^*∗*^	.06 (1.00)

Mesiodistal width of second premolar	7.15 ± 0.48	7.36 ± 0.50	7.11 ± 0.49	7.32 ± 0.50	7.02 ± 0.45	7.29 ± 0.50	C-CS	.04 (.82)	.04 (1.00)
							C-IS	.14 (.00)^*∗∗*^	.07 (.29)
							CS-IS	.09 (.06)	.04 (1.00)

Mesiodistal width of first molar	10.40 ± 0.59	10.81 ± 0.63	10.34 ± 0.61	10.74 ± 0.65	10.33 ± 0.50	10.73 ± 0.68	C-CS	.06 (.23)	.07 (1.00)
							C-IS	.07 (.15)	.08 (.25)
							CS-IS	.15 (1.00)	.01 (1.00)

Crowding	1.30 ± 1.48	6.57 ± 2.13	1.16 ± 1.58	5.99 ± 1.95	0.52 ± 1.41	5.72 ± 2.23	C-CS	−.02 (1.00)	.58 (1.00)
							C-IS	.78 (.01)^*∗*^	.86 (.02)^*∗*^
							CS-IS	.64 (.07)	−.27 (1.00)

Mild group (crowding < 3 mm); severe group (crowding > 4.5 mm); mesiodistal width of teeth, *n* = 40; crowding, *n* = 20; *p* values were calculated by RM-ANOVA; ^*∗*^*p* < .05; ^*∗∗*^*p* < .005.

**Table 4:** Comparison of linear measurements between mild and severe crowding in mandibular dentition (mm).

	Cast (C)		Cast scan (CS)		Intraoral scan (IS)		Mean difference (*p* value)		
	(mean ± SD)		(mean ± SD)		(mean ± SD)				
	Mild group	Severe group	Mild group	Severe group	Mild group	Severe group		Mild group	Severe group
Mesiodistal width of central incisor	5.62 ± 0.40	5.72 ± 0.39	5.63 ± 0.36	5.67 ± 0.37	5.64 ± 0.38	5.69 ± 0.37	C-CS	−.01 (1.00)	.04 (1.00)
							C-IS	−.02 (.1.00)	.03 (.62)
							CS-IS	−.01 (1.00)	−.01 (1.00)

Mesiodistal width of lateral incisor	6.30 ± 0.44	6.38 ± 0.41	6.38 ± 0.46	6.37 ± 0.34	6.24 ± 0.49	6.35 ± 0.41	C-CS	−.02 (1.00)	.01 (1.00)
							C-IS	.06 (.35)	.03 (.46)
							CS-IS	.04 (.99)	.02 (1.00)

Mesiodistal width of canine	7.13 ± 0.46	7.23 ± 0.40	7.10 ± 0.47	7.23 ± 0.40	7.10 ± 0.47	7.23 ± 0.42	C-CS	.03 (.48)	.01 (1.00)
							C-IS	.02 (1.00)	.01 (1.00)
							CS-IS	−.00 (1.00)	−.01 (1.00)

Mesiodistal width of first premolar	7.53 ± 0.44	7.76 ± 0.47	7.47 ± 0.45	7.66 ± 0.46	7.42 ± 0.48	7.66 ± 0.47	C-CS	.06 (.02)^*∗*^	.11 (.33)
							C-IS	.12 (.00)^*∗∗*^	.10 (.01)^*∗*^
							CS-IS	.05 (.42)	−.00 (1.00)

Mesiodistal width of second premolar	7.52 ± 0.52	7.63 ± 0.49	7.43 ± 0.55	7.56 ± 0.46	7.33 ± 0.57	7.57 ± 0.48	C-CS	.09 (.01)^*∗*^	.07 (1.00)
							C-IS	.19 (.00)^*∗∗*^	.06 (.00)^*∗∗*^
							CS-IS	.10 (.03)^*∗∗*^	−.01 (1.00)

Mesiodistal width of first molar	11.59 ± 0.57	11.57 ± 0.62	11.49 ± 0.58	11.55 ± 0.65	11.49 ± 0.60	11.53 ± 0.65	C-CS	.11 (.00)^*∗∗*^	.03 (1.00)
							C-IS	.10 (.00)^*∗∗*^	.04 (1.00)
							CS-IS	−.01 (1.00)	.01 (1.00)

Crowding	1.47 ± 1.04	6.68 ± 3.00	1.10 ± 0.94	6.06 ± 3.16	0.92 ± 1.01	6.18 ± 2.96	C-CS	.37 (.10)	.63 (1.00)
							C-IS	.55 (.06)	.51 (.02)^*∗*^
							CS-IS	.18 (1.00)	−.12 (1.00)

Mild group (crowding < 3 mm); severe group (crowding > 4.5 mm); mesiodistal width of teeth, *n* = 40; crowding, *n* = 20; *p* values were calculated by RM-ANOVA; ^*∗*^*p* < .05; ^*∗∗*^*p* < .005.

**Table 5:** Effect of severity of crowding on the dental measurements in the three dental models.

Measurement	Maxilla (*p* value)	Mandible (*p* value)
Mesiodistal width of central incisor	0.49	0.51
Mesiodistal width of lateral incisor	0.27	0.85
Mesiodistal width of canine	0.66	0.85
Mesiodistal width of first premolar	0.64	0.59
Mesiodistal width of second premolar	0.70	0.27
Mesiodistal width of first molar	0.96	0.62
Posterior sectional arch length	0.66	0.59
Anterior sectional arch length	0.80	0.82
Crowding	0.67	0.75

Mesiodistal width of teeth, *n* = 40; posterior sectional arch length, anterior sectional arch length, and crowding, *n* = 20; *p* values were calculated by RM-ANOVA with Greenhouse-Geisser correction.
